# Low‐Dose Lemon Myrtle Supplementation Enhances Muscle Hypertrophy in Older Adults Undergoing Low‐Load Resistance Training: A Randomized Controlled Trial

**DOI:** 10.1155/jare/8887586

**Published:** 2026-02-01

**Authors:** Risa Mitsuhashi, Shuji Sawada, Azusa Nishino, Shinichi Honda, Yuji Tominaga, Shiori Makio, Hayao Ozaki, Shuichi Machida

**Affiliations:** ^1^ Japanese Center for Research on Women in Sport, Juntendo University, Chiba, Japan, juntendo.ac.jp; ^2^ Faculty of Health and Sports Science, Juntendo University, Chiba, Japan, juntendo.ac.jp; ^3^ Kaneka Corporation, Osaka, Japan, kaneka.co.jp; ^4^ Graduate School of Medicine, Juntendo University, Tokyo, Japan, juntendo.ac.jp; ^5^ Department of Sport and Health Science, Tokai Gakuen University, Miyoshi, Aichi, Japan; ^6^ Graduate School of Health and Sports Science, Juntendo University, Chiba, Japan, juntendo.ac.jp

**Keywords:** lemon myrtle, muscle hypertrophy, plant extract, resistance training, sarcopenia

## Abstract

**Background/Objectives:**

Our previous study showed that a combination of lemon myrtle (LM) leaf extract at the conventional dose (250 mg/day, 2.5 mg/day as casuarinin) and low‐load resistance training using body weight led to significantly greater increases in muscle size than resistance training alone. This study aimed to determine whether LM supplementation at half the conventional dose (125 mg/day, 1.25 mg/day as casuarinin), combined with low‐load resistance training, could similarly enhance muscle hypertrophy in older adults and to evaluate the persistence of these effects during a detraining period.

**Methods:**

This was a randomized, placebo‐controlled, double‐blind, parallel‐group trial. Sixty Japanese men and women aged ≥ 65 years who were aware of age‐related declines in muscle strength participated. Participants were randomly assigned to a placebo group or an LM group (receiving 125 mg/day). Both groups performed low‐load, bodyweight resistance training twice weekly (three sets of four exercises). Anterior thigh muscle thickness was assessed before and after the 12‐week intervention, and after a subsequent 6‐week detraining period.

**Results:**

The LM group showed a significantly greater increase in anterior thigh muscle thickness than the placebo group. However, LM supplementation did not help maintain muscle hypertrophy during the detraining period.

**Conclusions:**

LM supplementation at half the conventional dose enhances skeletal muscle hypertrophy following 12 weeks of low‐load resistance training in older adults experiencing muscle strength decline.

**Trial Registration:** UMIN Clinical Trials Registry: UMIN000054801

## 1. Introduction

Population aging is a growing global concern. Maintaining skeletal muscle mass and function is essential for healthy aging, as skeletal muscles play a central role in physical mobility, activities of daily living [1], and whole‐body energy metabolism, including glucose and lipid regulation [2]. However, skeletal muscle mass and strength progressively decline with age [3, 4], a condition known as sarcopenia. Sarcopenia is associated with various chronic diseases [5–8], functional impairments [9, 10], and increased mortality [11]. Recognizing its clinical importance, sarcopenia was assigned a diagnostic code (M62.84) in the International Classification of Diseases, 10th Revision, in 2016.

Recent guidelines from the European and Asian Working Groups on Sarcopenia have emphasized the importance of combined interventions involving exercise and nutrition for preventing and treating sarcopenia [12, 13]. Resistance training and sufficient protein intake are especially effective strategies to maintain muscle health in older adults [14]. Several interventional studies have demonstrated that resistance training combined with nutritional supplementation, such as whey protein or branched‐chain amino acids, leads to improved muscle mass and function in both healthy individuals and those with sarcopenia [15–18].

While protein‐based supplementation is widely studied, there is increasing interest in plant‐derived bioactive compounds for their potential to promote muscle health. Lemon myrtle (LM), a plant from the Backhousia genus of the Myrtaceae family, possesses antimicrobial [19, 20], anti‐inflammatory [21–23], and antioxidant properties [22–25]. Our previous study showed that supplementation with LM leaf extract (250 mg/day, 2.5 mg/day as casuarinin) significantly enhanced skeletal muscle hypertrophy when combined with low‐load, bodyweight resistance training in older adults, compared to exercise alone [26]. Furthermore, LM extract and its active compound, casuarinin, were found to activate skeletal muscle satellite cells (SCs) in vitro and in vivo [27]. Given that SC dysfunction is implicated in sarcopenia [28], these findings suggest a potential role for LM in mitigating age‐related muscle decline.

Importantly, the feasibility and accessibility of interventions are crucial when targeting older adults. Low‐load, bodyweight resistance training is a practical approach for this population. Additionally, lower doses of functional foods or supplements, if effective, could enhance compliance and reduce costs. However, whether lower doses are as effective as those used in previous studies remains unclear, given the limited understanding of dose–response relationships for such plant‐based interventions.

Although higher volumes of resistance training have been associated with greater muscle hypertrophy [29], the optimal combination of training volume and supplement dosage, particularly in frail or aging populations, is not well established. Our pilot findings using the conventional LM dose were promising but limited by sample size [26].

Therefore, in the present study, we aimed to determine whether a lower dose of LM supplementation (125 mg/day as LM extract, 1.25 mg/day as casuarinin), combined with a typical bodyweight resistance training program, could still produce significant increases in muscle size among older adults. Furthermore, this study examined whether LM supplementation could influence the maintenance of muscle size during a subsequent detraining period. These findings highlight the potential of low‐dose, plant‐derived supplementation as a practical and scalable adjunct to exercise‐based interventions for preventing sarcopenia and promoting muscle health.

## 2. Materials and Methods

### 2.1. Participants

Older Japanese men and women were recruited through a Contract Research Organization. Inclusion criteria were as follows: (1) age ≥ 65 years and (2) self‐reported decline in muscle mass or strength, as indicated by perceived slowing of maximum walking speed. Exclusion criteria included the following: (1) use of antidiabetic medications; (2) medical contraindications to exercise; (3) symptoms interfering with training (e.g., lower back pain or arthralgia); (4) regular use of supplements, functional foods, or medications for musculoskeletal health; (5) daily attendance at a fitness club; (6) evident undernutrition; and (7) deemed ineligible by a physician.

All participants received detailed explanations about the study’s purpose, procedures, and potential risks, and provided written informed consent. Initial screening (medical examination, blood test, and questionnaire) was conducted on 244 consenting individuals. Based on findings such as arrhythmia, shortness of breath, heart disease history, hypertension, obesity (BMI > 30 kg/m^2^), potential diabetes (elevated blood glucose or HbA1c), underweight (BMI < 18.5 kg/m^2^), and regular exercise habits, 128 individuals were excluded.

A total of 116 participants underwent a second screening (body composition, anterior thigh [AT] muscle thickness, and 30‐s chair stand [CS‐30] test). Those with AT muscle thickness ≥ 35.0 mm in men or ≥ 30.0 mm in women were excluded for having no evident decline in physical function. Ultimately, 60 Japanese adults (mean age: 70.3 ± 4.8 years) were included and randomly allocated to two groups using stratified randomization based on age, sex, AT muscle thickness, and CS‐30 score.

### 2.2. Study Design

A randomized, double‐blind, placebo‐controlled, parallel‐group trial was conducted to evaluate the efficacy of LM extract. The study adhered to the Declaration of Helsinki and was approved by the Ethics Committee of the Juntendo University Graduate School of Health and Sports Science (2024–51)

Participants were randomly assigned to receive either a placebo or LM supplement (125 mg/day as LM extract, 1.25 mg/day as casuarinin), alongside a typical‐volume low‐load resistance training program (three sets, twice weekly). Blinded assessors measured outcomes at baseline (Week 0), midintervention (Week 6), postintervention (Week 12), and after a subsequent 6‐week detraining period (Week 18). The primary outcome was AT muscle thickness.

All training sessions were conducted by certified instructors from Sports Oasis Inc. Participants were instructed to maintain their usual diet and lifestyle throughout the intervention. Dietary intake was assessed at Weeks 0, 6, 12, and 18 using the Brief‐type Self‐administered Diet History Questionnaire (BDHQ).

### 2.3. Resistance Training

Participants engaged in bodyweight‐based resistance training twice weekly for 12 weeks at the Sports Oasis Inc., Yukigaya branch. The training program followed the protocol from our previous study [24] and included squats, split squats (right and left), push‐ups, and crunches. Exercise order, repetitions, and rest intervals are summarized in Table 1. For the first 1–2 weeks, all exercises were performed at 6 repetitions × 2 sets. For Weeks 3–4, all exercises were performed at 8 repetitions × 2 sets. For Weeks 5–6, only the split squat was performed at 8 repetitions × 2 sets, while the other exercises were performed at 8 repetitions × 3 sets. From Week 7 onward, all exercises were performed at 8 repetitions × 3 sets.

**TABLE 1:** Training program.

Week	1–2		3–4		5–6		7–8		9–10		11–12	
Exercises	Order	Sets	Order	Sets	Order	Sets	Order	Sets	Order	Sets	Order	Sets
Squat	1	2	1	2	1	3	1	3	3	3	3	3
Split squat (right leg)	2	2	2	2	2	2	2	3	1	3	1	3
Split squat (left leg)	3	2	3	2	3	2	3	3	2	3	2	3
Push‐up	4	2	4	2	4	3	4	3	4	3	4	3
Crunch	5	2	5	2	5	3	5	3	5	3	5	3

Repetitions per set	6		8		8		8		8		8	
CON‐ECC (seconds)	3‐3		3‐3		3‐3		3‐3		3‐3		3‐3	
Interval (seconds)	60		60		60		60		60		60	
Frequency (days/week)	2		2		2		2		2		2	

*Note:* CON‐ECC, CON‐ECC; the time (seconds) in the concentric phase and eccentric phase of each repetition. Interval: rest time between each set.

### 2.4. Test Food

The LM extract (Lemon myrtle UP, Kaneka Corporation, Osaka, Japan) was produced by hot‐water extraction and powderized with dextrin. Each tablet contained 125 mg of LM extract and 1.25 mg of casuarinin, along with excipients (crystalline cellulose, maltitol, silicon dioxide, calcium stearate). Placebo tablets were identical in appearance and contained only excipients and caramel coloring.

Participants took one tablet daily with water for 18 weeks, typically in the morning. Compliance was assessed using participants’ daily records and by verifying the number of returned empty supplement packages.

### 2.5. Body Composition

Body weight, fat mass, and body fat percentage were assessed using a bioelectrical impedance analyzer (InBody 270, InBody Japan Inc., Tokyo, Japan). BMI was calculated as weight (kg) divided by height squared (m^2^).

### 2.6. Muscle Thickness

AT muscle thickness was measured using B‐mode ultrasound (5–18 MHz, LOGIQ e, GE HealthCare Japan). Measurements were taken at the midpoint between the greater trochanter and lateral condyle of the femur, with participants resting in a supine position after sitting for 30 min. The procedure followed the method described by Ozaki et al. [30]. All measurements were performed by a single researcher with more than 10 years of experience in ultrasound assessment. Test–retest (intersession) reliabilities were calculated using the intraclass correlation coefficient (ICC), standard errors of measurement (SEMs), and minimal difference (MD). The ICC, SEM, and MD for AT muscle thickness determined in 10 older subjects were 0.992, 0.37, and 1.03 mm, respectively.

### 2.7. Physical Functions

Physical function was assessed using the 10‐m walk and CS‐30 tests. Walking speed was measured on a 10‐m hard‐surface track (1 m wide), with two trials conducted at maximum effort.

The CS‐30 test involved performing as many sit‐to‐stand movements as possible in 30 s from a 40‐cm‐high chair, with arms crossed over the chest, as described by Jones et al. [31].

### 2.8. BDHQ

The BDHQ, a validated tool in Japan, was used to assess dietary habits over the past month. The questionnaire covered food frequency and intake patterns and took approximately 15 min to complete. Nutrient intakes were calculated using standard Japanese food composition tables.

### 2.9. Safety Assessment

Participants kept daily logs of symptoms, medication use, supplement intake, and lifestyle habits. These were reviewed in detail by study staff every 6 weeks. Medical interviews, blood pressure, pulse, and blood tests were conducted before and after the intervention.

### 2.10. Statistical Analyses

Data are presented as means ± standard deviations. Baseline characteristics were compared using Fisher’s exact test or unpaired *t*‐tests. Between‐group comparisons for percentage changes in AT thickness, walking speed, and CS‐30 scores were analyzed using a closed testing procedure. Independent unpaired Student′s *t*‐tests were used for group comparisons at 12 weeks, and if significant (*p* < 0.05), also at 6 weeks. To examine the effects of LM intake during the 6‐week detraining period (Week 18) following the training intervention (Week 12), unpaired Student’s *t*‐tests were conducted in an exploratory manner. Adverse event incidence was assessed using Fisher’s exact test. All statistical analyses were conducted using BellCurve for Excel (Social Survey Research Information Co., Ltd., Japan), with significance set at *p* < 0.05.

## 3. Results

**FIGURE 1:**
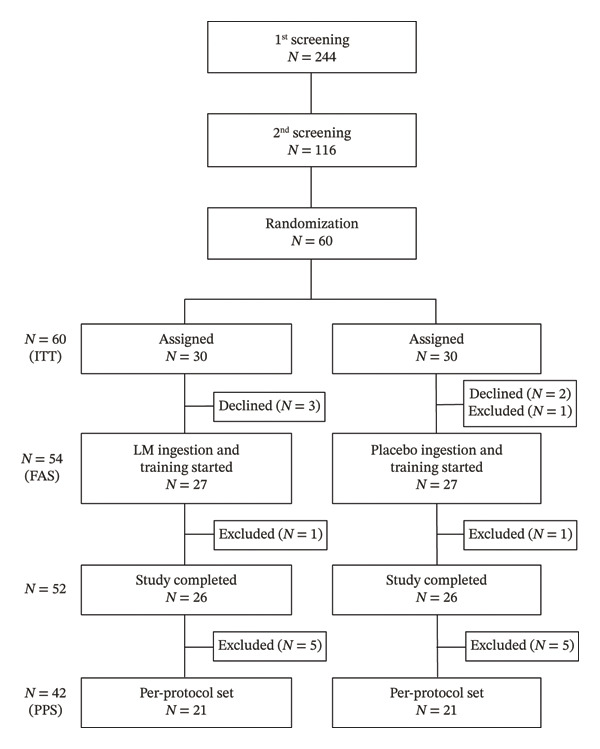
Flowchart showing the distribution of participants throughout the intervention.

The participant flowchart is shown in Figure 1. A total of 244 older adults were screened, and 60 participants met the eligibility criteria based on the investigators’ inclusion and exclusion criteria. These participants were randomly assigned to either the LM group (*n* = 30) or the placebo group (*n* = 30).

Before the intervention, five participants withdrew for personal reasons, and one participant was excluded by the principal investigators due to an adverse event deemed unrelated to either the test food or resistance training. During the intervention, two participants were excluded by the principal investigators due to an adverse event deemed unrelated to either the test food or resistance training and due to compliance violations, respectively. As a result, 52 participants (26 in each group) completed the 12‐week intervention.

However, 10 participants (five from each group) were excluded from the statistical analyses due to protocol violations, such as engaging in unsupervised exercise, missing more than three training sessions or two consecutive sessions, significant lifestyle changes, or subsequently meeting exclusion criteria. These exclusions were made while maintaining blinding of group allocation.

Thus, 42 participants (21 in the LM group and 21 in the placebo group) were included in the final statistical analyses. The test food compliance rate in both groups exceeded 99%.

### 3.1. Baseline Characteristics

Baseline characteristics of the participants are presented in Table 2. No significant differences were found between the LM and placebo groups in terms of sex distribution, age, body weight, height, or BMI. Additionally, no significant differences were observed in AT muscle thickness or physical function measures (normal and maximum walking speeds and CS‐30 scores) at baseline.

**TABLE 2:** Baseline characteristics of the participants.

	**LM (*n* = 21)**	**Placebo (*n* = 21)**	**p** ‐value**^1^**

Participants (number) [men:women]	21 [11:10]	21 [14:7]	0.530
Physical parameters			
Age (year)	70.3 ± 4.8	71.3 ± 5.4	0.550
Body weight (kg)	58.48 ± 10.21	57.92 ± 5.67	0.828
Height (cm)	160.85 ± 8.41	162.48 ± 8.09	0.527
BMI (kg/m2)	22.43 ± 2.22	21.99 ± 2.06	0.507
Skeletal muscle mass (kg)	23.24 ± 5.55	23.41 ± 3.25	0.903
Skeletal muscle mass index (kg/m^2^)	6.51 ± 1.17	6.68 ± 0.63	0.567
Muscle thickness			
Anterior thigh (mm)	25.78 ± 4.74	25.90 ± 3.35	0.923
Physical functions			
Normal walking speed (m/sec)	1.45 ± 0.2	1.47 ± 0.17	0.850
Maximum walking speed (m/sec)	2.09 ± 0.37	2.00 ± 0.26	0.348
CS‐30 (times)	20.9 ± 6.3	19.2 ± 5.4	0.348
Food intake			
Energy (kcal/day)	1806.47 ± 691.52	1530.77 ± 482.07	0.142
Protein (g/day)	73.90 ± 31.88	66.00 ± 30.41	0.416
Fat (g/day)	60.68 ± 24.90	52.61 ± 19.31	0.248
Carbohydrates (g/day)	230.43 ± 90.46	183.00 ± 61.76	0.054

*Note:* Values are presented as the means ± SD

^1^Comparison between groups using Fisher’s exact test or unpaired Student’s *t*‐test.

Furthermore, dietary intake assessed by the BDHQ showed no significant differences in daily total energy, protein, fat, or carbohydrate intake between the two groups prior to the intervention.

### 3.2. Postintervention Outcomes

Participants completed a 12‐week intervention consisting of typical‐volume resistance training (three sets, twice per week) in combination with either the LM supplement or a placebo. They also completed a subsequent 6‐week detraining period with either the LM supplement or a placebo intake. The changes in AT muscle thickness and physical function (normal and maximum walking speeds, CS‐30 scores) are summarized in Table 3.

**TABLE 3:** Effects of resistance training and the LM supplement on muscle thickness and physical function.

	**Time**	**LM (*n* = 21)**	**Placebo (*n* = 21)**	**p** ‐value**^1^**	**Effect size**

*Muscle thickness*					
Anterior thigh	0 weeks	25.8 ± 4.74	25.9 ± 3.35	0.923	0.031
	12 weeks	28.8 ± 5.98	27.5 ± 3.39	0.380	0.281
	18 weeks	26.8 ± 4.92	26.8 ± 3.21	0.982	0.007

*Physical functions*					
Normal walking speed	0 weeks	6.5 ± 1.66	6.8 ± 1.13	0.541	0.195
	12 weeks	6.5 ± 1.60	6.7 ± 1.05	0.588	0.173
	18 weeks	6.4 ± 1.54	6.6 ± 1.05	0.546	0.193

Maximum walking speed	0 weeks	4.9 ± 0.77	5.1 ± 0.67	0.422	0.256
	12 weeks	4.7 ± 0.91	4.8 ± 0.76	0.558	0.187
	18 weeks	4.7 ± 0.86	4.7 ± 0.86	0.558	0.190

CS‐30	0 weeks	20.9 ± 6.32	19.2 ± 5.35	0.476	0.300
	12 weeks	24.7 ± 6.73	22.4 ± 5.98	0.252	0.368
	18 weeks	25.3 ± 6.82	22.2 ± 5.88	0.138	0.480

*Note:* Values are presented as the means ± SD.

^1^Comparison between groups using the unpaired Student’s *t*‐test.

**FIGURE 2:**
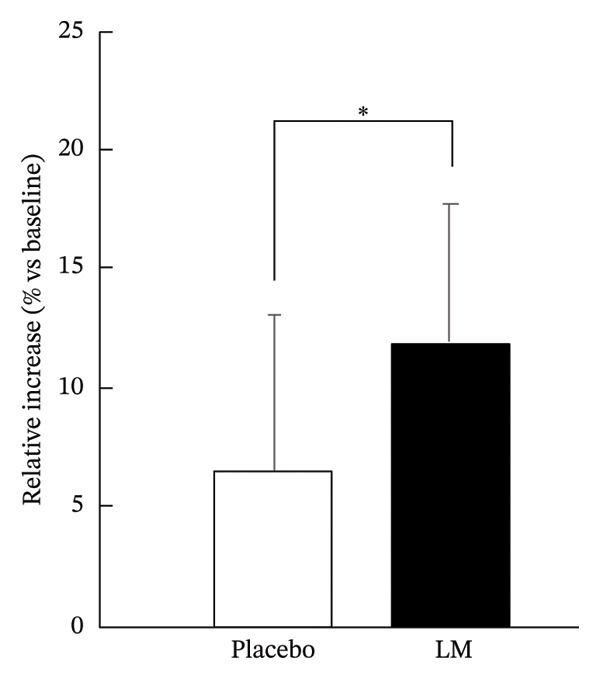
The changes in AT muscle thickness after the 12‐week intervention. Values are presented as the means ± SD. Comparison between groups using the unpaired Student’s *t*‐test (^∗^: *p* < 0.05).

While both groups showed similar improvements in physical function after 12 weeks, the relative increase in AT muscle thickness was significantly greater in the LM group (11.9%) than in the placebo group (6.5%) (*p* < 0.05, Figure 2). However, LM supplementation did not contribute to the maintenance of muscle hypertrophy during the detraining period (Table 3).

BDHQ‐based dietary assessments revealed no significant differences in daily total energy, protein, fat, or carbohydrate intake between the LM and placebo groups before or after the intervention (data not shown).

### 3.3. Safety Assessment

The number of adverse events reported during the 12‐week intervention and the subsequent 6‐week detraining period was comparable between the LM group (*n* = 15) and the placebo group (*n* = 13), with no significant difference. All adverse events were deemed by study physicians to be unrelated to the test food and resistance training.

## 4. Discussion

This study demonstrated that supplementation with a low dose (125 mg/day) of LM extract, 1.25 mg/day as casuarinin, when combined with low‐load, bodyweight resistance training, significantly enhanced AT muscle hypertrophy in older adults compared to training alone. These findings extend our previous work [26], which showed similar benefits using a higher dose (250 mg of LM extract/day, 2.5 mg as casuarinin/day), and provide novel evidence that even half the conventional dose can be effective when paired with appropriate exercise. Importantly, both groups followed an identical resistance training regimen with comparable improvements in physical function, yet only the LM group showed a significantly greater increase in muscle thickness. This indicates that the observed hypertrophic effect can be attributed to the LM supplementation rather than differences in training volume or intensity. However, LM supplementation with a low dose did not contribute to the maintenance of muscle hypertrophy during the detraining period.

The magnitude of hypertrophy observed in this study (> 10%) may appear relatively large for bodyweight resistance training; however, previous studies support its plausibility. A dose–response relationship between training volume and hypertrophy has been reported [29], and our recent randomized controlled trial using the same exercise protocol showed a 9.7% increase in AT muscle thickness when performed at 3 sets (6–8 repetitions per set), compared with 5.4% when performed at 1 set in older adults [32]. Additionally, another of our previous studies using more training volume (3 sets of 8–15 repetitions per set) has demonstrated that a 12‐week intervention of lower body training resulted in a 13% increase [30], whereas Fujita et al. (2016) reported a 6.2% increase in muscle thickness following 12 weeks of once‐weekly bodyweight squat training in frail older adults. Collectively, these findings indicate that bodyweight resistance training can induce exercise dose‐dependent hypertrophy in older adults and support the plausibility of the hypertrophic response in the present study [33].

Although the LM group exhibited significantly greater hypertrophy, no corresponding improvements were detected in physical function. This apparent dissociation between muscle size and performance may be explained by two factors. First, the participants were physically independent older adults with relatively high baseline functional scores, suggesting the possibility of a ceiling effect that limited the detectability of further improvements. Second, functional capacity depends not only on morphological adaptations but also on neural activation, coordination, and task‐specific motor learning, which may not progress in parallel with hypertrophic responses [34, 35]. Therefore, increases in muscle size alone may not always translate to measurable improvements in functional outcomes.

### 4.1. Practical Implications for Sarcopenia Prevention

Importantly, from a public health perspective, the feasibility of combining low‐load resistance training with a minimal‐dose, plant‐based supplement becomes particularly relevant in community settings where access to specialized exercise equipment or dietary counseling may be limited. In contrast, many existing nutritional strategies rely on relatively high protein or amino acid intake, which may not be well tolerated or preferred by all individuals [36]. In contrast, LM is a plant‐derived extract that was well tolerated, with no serious adverse events and a high compliance rate (> 99%), suggesting good feasibility for long‐term use. These findings support the potential of LM as part of a multimodal strategy to prevent or mitigate sarcopenia in aging populations, particularly among frail or nutritionally vulnerable older adults who are unable or unwilling to follow conventional high‐intensity or high‐nutrient protocols.

### 4.2. Possible Mechanisms of Action

Although the exact mechanisms were not directly assessed in this trial, previous research has identified casuarinin, a key ellagitannin component of LM extract, as a potential bioactive compound promoting SC activation [27]. SC dysfunction is a known contributor to impaired muscle regeneration in aging [37]. It is plausible that LM potentiates the anabolic environment induced by resistance training, facilitating hypertrophic processes even at low doses. Future mechanistic studies are warranted to verify this hypothesis.

### 4.3. Comparison With Other Plant‐Derived Supplements

While the field of sports nutrition has traditionally focused on protein‐ or amino acid–based supplements, there is growing interest in botanical ingredients with anti‐inflammatory and antioxidant effects that support muscle health [38]. Our findings contribute to this emerging area by showing that LM, a supplement derived from a culinary and medicinal plant native to Australia, can enhance training‐induced hypertrophy. To our knowledge, this is the first study to show that a plant extract can augment resistance training outcomes in older adults at such a low dose.

### 4.4. Strengths and Limitations

A major strength of this study is the double‐blind, placebo‐controlled design with high adherence and a well‐characterized training program. Moreover, dietary intake was carefully monitored, and no significant between‐group differences were observed, minimizing the confounding effects of nutrition. Importantly, unlike conventional protein or amino acid supplementation, which may suppress subsequent food intake due to their satiating effects [39], the LM supplementation used in this study did not affect overall dietary intake. This suggests that LM, even when administered daily, does not interfere with natural food consumption or caloric intake—a particularly relevant advantage in older adults who may already experience diminished appetite or nutritional risk. This feature may allow LM to be used complementarily, without compromising regular meals or dietary variety. Furthermore, the extremely low daily dose (125 mg/day) required to achieve a measurable anabolic effect not only enhances feasibility but also reduces the risk of gastrointestinal discomfort often reported with higher dose nutritional supplements. Therefore, LM supplementation represents a novel strategy for augmenting resistance training adaptations in older adults without the common nutritional trade‐offs associated with traditional ergogenic aids.

However, the study has some limitations. First, the sample size, while adequate for detecting group differences in muscle thickness, may be underpowered for assessing secondary outcomes such as walking speed or functional performance. Second, the intervention period was limited to 12 weeks, and the long‐term sustainability of the effect remains to be confirmed. Third, mechanistic outcomes (e.g., SC activity, muscle protein synthesis) were not directly measured. Fourth, although differences in dietary intake between groups were not statistically significant, future studies should control or standardize dietary intake to minimize potential confounding.

### 4.5. Future Directions

Future studies should explore the dose–response relationship of LM supplementation in more detail, including even lower doses and different administration durations. Additionally, studies combining LM with other exercise modalities (e.g., aerobic training or neuromuscular stimulation) or nutritional interventions (e.g., protein intake modulation) may further clarify its potential in comprehensive sarcopenia prevention programs. Biomarker assessments and muscle biopsies could also help clarify the molecular mechanisms underlying the observed effects.

## 5. Conclusions

In conclusion, LM supplementation at half the conventional dose significantly enhanced muscle hypertrophy in older adults undergoing low‐load resistance training. These findings support the use of low‐dose, plant‐based interventions as scalable, well‐tolerated strategies for preserving muscle mass in aging populations.

## Funding

This work was supported by Kaneka Corporation​ (Osaka, Japan).

## Conflicts of Interest

Azusa Nishino, Shinichi Honda, and Yuji Tominaga are employees of Kaneka Corporation (Osaka, Japan), which funded this study. They were responsible for the test supplements but were not involved in the investigation, formal analysis, or data curation. The results of this study are clear and without fabrication, falsification, or inappropriate data manipulation.

## Data Availability

Data supporting the findings of this study are available upon request from the corresponding author.
